# Application of indocyanine green fluorescence imaging in hepatobiliary surgery

**DOI:** 10.1097/JS9.0000000000001802

**Published:** 2024-06-17

**Authors:** Jia Zhou, Zhiguo Tan, Bo Sun, Yufang Leng, Sulai Liu

**Affiliations:** a Department of Hepatobiliary Surgery/Central Laboratory, Hunan Provincial People’s Hospital (The First Affiliated Hospital of Hunan Normal University), Changsha, Hunan Province; b Department of Anesthesiology, The First School of Clinical Medicine, Lanzhou University, Lanzhou; c Hunan Engineering Research Center of Digital Hepatobiliary Medicine, Changsha; d Hunan Provincial Key Laboratory of Biliary Disease Prevention and Treatment, Changsha, People’s Republic of China

**Keywords:** fluorescence imaging, hepatobiliary surgery, indocyanine green, liver resection

## Abstract

Indocyanine green (ICG) is a fluorescent dye with an emission wavelength of about 840 nm, which is selectively absorbed by the liver after intravenous or bile duct injection, and then it is excreted into the intestines through the biliary system. With the rapid development of fluorescence laparoscopy, ICG fluorescence imaging is safe, feasible, and widely used in hepatobiliary surgery. ICG fluorescence imaging is of great significance in precise preoperative and intraoperative localization of liver lesions, real-time visualization of hepatic segmental anatomy, intrahepatic and extrahepatic biliary tract visualization, and liver transplantation. ICG fluorescence imaging facilitates efficient intraoperative hepatobiliary decision-making and improves the safety of minimally invasive hepatobiliary surgery. Advances in imaging systems will increase the use of fluorescence imaging as an intraoperative navigation tool, improving the safety and accuracy of open and laparoscopic/robotic hepatobiliary surgery. Herin, we have reviewed the status of ICG applications in hepatobiliary surgery, aiming to provide new insights for the development of hepatobiliary surgery.

## Introduction

Highlights

- A more accurate visualization technique in the field of hepatobiliary surgery.
- A safe, efficient, and feasible advance technique in precise preoperative and intraoperative localization of liver lesions, real-time visualization of hepatic segmental anatomy, intrahepatic and extrahepatic biliary tract visualization, and liver transplantation;
- Advances in imaging systems will increase the use of fluorescence imaging as an intraoperative navigation tool, improving the safety and accuracy of open and laparoscopic/robotic hepatobiliary surgery.

The application of digital medical imaging technology in hepatobiliary surgery has changed the traditional concept of liver surgery, creating a pioneering field in precision surgery, and bringing more effective diagnosis and management^1,2^. The key issues in the surgical treatment of hepatobiliary lesions are accurate localization, resection, and reconstruction with the preservation of hepatic parenchyma and function. Tumor visualization, anatomical hepatectomy, biliary visualization, and hepatic resection margin definition are essential to preserve the liver parenchyma and reduce postoperative complications and mortality^3^. In the 1980s, the development of intraoperative ultrasound combined with endoscopy enabled surgeons to observe tumors in real-time intraoperatively, improving the accuracy of tumor localization^4,5^. In recent years, fluorescent dye-based imaging has further improved surgeons’ ability to visualize liver lesions in real-time and has been widely used in the field of hepatobiliary surgery^6–8^.

Indocyanine green (ICG) is a water-soluble dye that binds to albumin, distributing rapidly and uniformly in the bloodstream, undergoing hepatic metabolism, and biliary excretion^9^. Its physicochemical properties allow it to be excited by near-infrared light with a wavelength of 750–810 nm, emitting fluorescence around 840 nm, with tissue penetration depth ranging from 5 to 10 mm^10,11^. Since the U.S. Food and Drug Administration (FDA) approved clinical trials in the 1950s, ICG was initially utilized in hepatic function diagnostic tests^9^. In 2008, Aoki *et al*.^12^ introduced ICG fluorescence imaging technology as a novel navigation tool in liver resection surgery. Subsequently, laparoscopic fluorescence imaging has rapidly evolved, and ICG fluorescence imaging technology has been widely applied in hepatobiliary surgical procedures^13^. Currently, clinical societies, including the International Society for Fluorescence-Guided Surgery (ISFGS), support the application of fluorescence-guided surgery in hepatobiliary surgery and other specialties through clinical practice and research. Since its introduction in liver surgery, ICG fluorescence imaging technology has demonstrated significant clinical value and potential applications in liver tumor staining, anatomical liver resections, intrahepatic and extrahepatic biliary imaging, liver transplantation, etc.

Here, we summarize the applications of ICG in hepatobiliary surgery, which provide a new perspective and a more accurate visualization technique of ICG in hepatobiliary surgery.

## Overview of ICG in intraoperative fluorescence imaging

**Figure 1:**
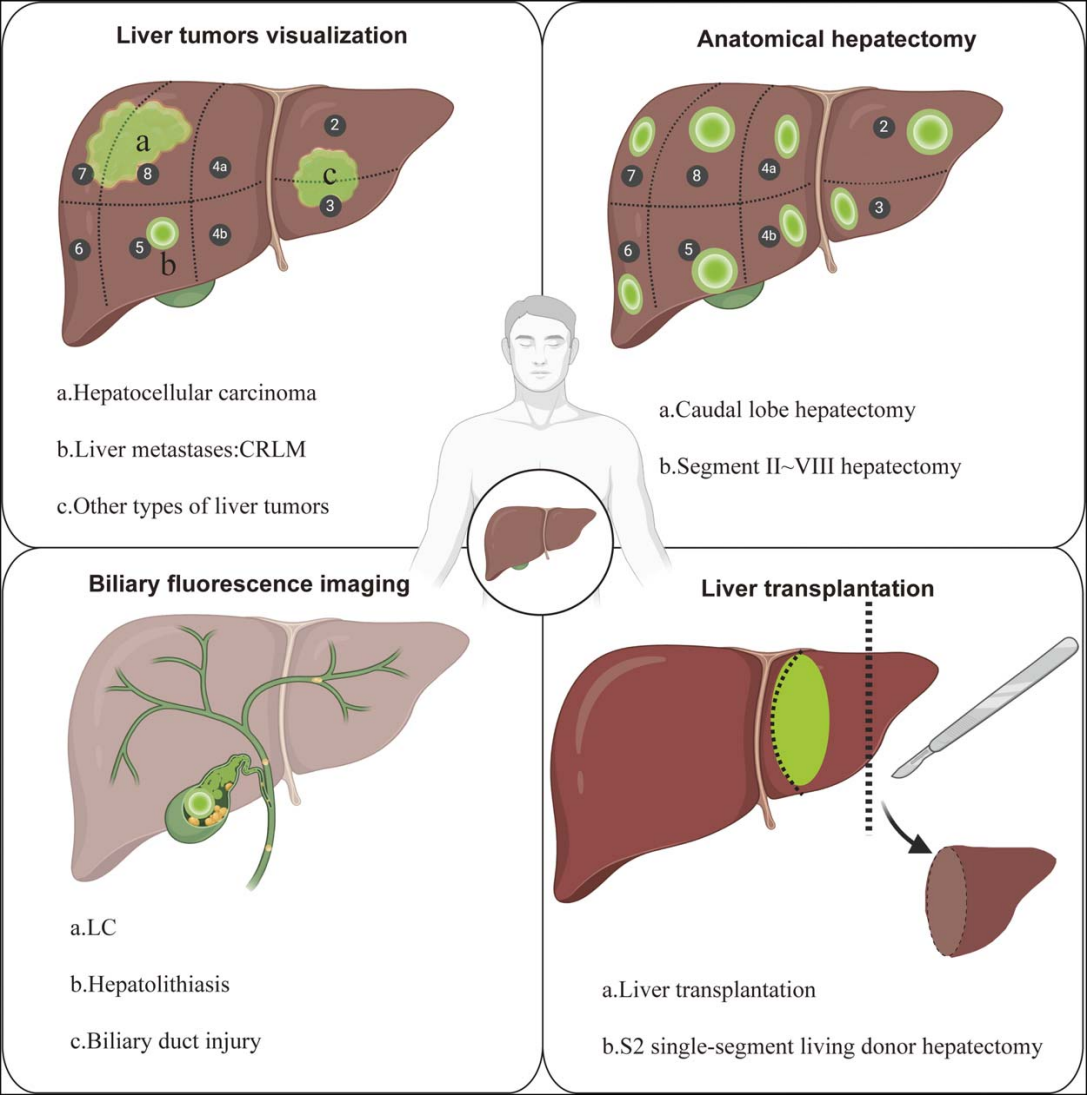
Overview of clinical applications of fluorescence-guided surgery in liver tumors, anatomical hepatectomy, biliary surgery, and liver transplantation. CRLM, colorectal cancer liver metastases; LC, laparoscopic cholecystectomy.

ICG is a disulfonated indocyanine dye that rapidly binds to plasma proteins and lipoproteins in a physiological environment and is rapidly distributed throughout the systemic vasculature with the blood circulation^10^. ICG is mainly metabolized by the liver and excreted through the biliary system and does not participate in enterohepatic circulation^10^. The half-life of ICG is 3–4 min, and it can be completely metabolized within 10–20 min after entering the bloodstream^11^. ICG has no adverse effects at a concentration of less than 2.0 mg/kg and can be visualized in vivo through local or intravenous injection. ICG has an absorption spectrum in the infrared region, and it emits fluorescence when stimulated by infrared light^14^. Infrared light can also penetrate up to 1 cm of living tissue, allowing the optical information from ICG to be obtained beyond the tissue surface^15^. With the rapid development of laparoscopic fluorescence imaging, ICG fluorescence imaging has been widely used in hepatobiliary surgery, including visualization of the biliary tract, visualization of liver lesions, precise anatomical liver resection, and liver transplantation (Fig. 1).

For fluorescent cholangiography, ICG can be injected intravenously or intrabiliary^16^. In 2009, Ishizawa *et al*.^17^ first reported that the bile duct was immediately visualized by injecting 0.025 mg/ml ICG through the cystic duct. Ishizawa *et al*.^18,19^ found that the concentration of ICG in the biliary tract reached a peak at 2 h after intravenous injection. Therefore, 2.5 mg of ICG was injected intravenously at 1 h before laparoscopic cholecystectomy (LC). Compared with transcystic duct intubation angiography, intravenous ICG biliary visualization is safer and more convenient. In patients with preoperative endoscopic or percutaneous biliary drainage tubes, it is preferable to attempt fluorescence cholangiography by intraduct injection of ICG^16^. The optimal fluorescence imaging is a high fluorescence signal in the bile ducts and a low fluorescence signal in the liver tissue in the background. The dose and administration time of ICG are the key factors affecting the performance of high-quality fluorescence imaging. Recent studies have suggested that ICG can be injected 30–60 min before surgery at a dose of 0.05 mg/kg or a total dose of 2.5 mg^18,20^. However, other studies have confirmed that intravenous ICG administration within 24 h before surgery can improve the fluorescence ratio between the biliary tree and the liver parenchyma^21^. In addition, Pujo-Cano *et al*.^22^ reduced the dosage of ICG and intravenously injected 0.25 mg during anesthesia induction, which can also reduce the background fluorescence intensity of the liver and improve the contrast between the bile duct and liver. It has not been reported whether the rate of bile duct recognition after earlier or reduced dose administration is comparable to that with intravenous 2.5 mg ICG. In conclusion, intravenous injection of 2.5 mg is currently the most used route and dose for ICG fluorescence cholangiography.

For intraoperative visualization of liver tumors, the time of administration has been described. According to the literature report, the most commonly used dose of peripheral intravenous administration for negative staining is 2.5 mg, ranging from 0.025 to 25 mg^23^, while another study has found that reducing the dose of ICG to 0.5 mg during negative staining can reduce the penetration of ICG into the target liver segment through vascular branches^24^. Therefore, in practice, 0.25 mg ICG can be given first, and then an appropriate amount of ICG can be added according to the staining effect. Generally, the total amount of ICG can be significantly stained up to 0.5 mg. For ICG-positive staining, another study has pointed out that the common dose range of ICG is 0.025–12.5 mg^23^. In addition, ICG can be diluted and used according to the liver volume in the portal vein drainage area to be stained^25^. However, in the application of fluorescent staining of liver tumors, the dosage of ICG should be reduced as much as possible to avoid excessive ICG entering the systemic circulation and causing unnecessary effects. According to most studies, a dose of 0.5 mg/kg given within 14 days before surgery, especially within 3 days before surgery, is reported to be effective for intraoperative visualization of liver tumors. If the interval between administration and surgery is long, an additional dose of 0.02–0.5 mg per kilogram is required^16,23^. In patients with impaired liver function due to cirrhosis or other reasons, ICG injection should be avoided the day before surgery to minimize fluorescence signals in liver tissue in the background and false-positives^26,27^.

The administration time, injection ways, and injection dose of ICG vary according to the condition and the purpose of surgery. The recommendations of clinical research are also different. Therefore, this article summarizes the following based on an international consensus participated by Wang *et al*.^16^ (Table 1).

**Table 1:** The optimal usage of ICG in hepatobiliary surgery.

Procedure	Dose of ICG	Timing of administration
Laparoscopic cholecystectomy	0.05 mg ICG/kg body weight or 2.5 mg ICG administered intravenously	30–60 min preoperatively
Tumor imaging	0.5 mg ICG/kg body weight administered intravenously	10–14 days before surgery
Anatomic resection-negative staining	2.5 mg ICG administered intravenously	After ligation of the Glissonian pedicle to the tumor-bearing segment
Anatomic resection-positive staining	5 ml of ICG at a concentration of 0.05-0.025 mg ICG/ml aqueous solvent	Intraoperatively
LDLT-biliary mapping	2.5 mg ICG administered intravenously	Intraoperatively 15–30 min before dissecting the hilar plate

ICG, indocyanine green; LDLT, living donor liver transplantation.

## Application of ICG fluorescence imaging in hepatobiliary surgery

Although ICG imaging has been used to detect liver function and blood flow for more than 50 years^28^, it has been used in hepatobiliary surgery for only more than a decade. With the introduction of the Photo Dynamic Eye (PDE) fluorescence imaging system developed by Hamamatsu Photonic, Japan, ICG fluorescence imaging technology has achieved rapid development in hepatobiliary surgery^29^. Ishizawa *et al*.^26^ performed the first case of fluorescent cholangiography-guided LC in humans by intravenous injection of ICG in 2008 and first reported open hepatectomy by using ICG fluorescence visualization navigation technology in 2009. Subsequently, ICG fluorescence imaging technology was introduced into laparoscopic hepatectomy in 2012^13^. At present, fluorescence imaging technology has been successfully used in a variety of hepatobiliary surgeries, such as LC, anatomical hepatectomy, and liver transplantation. Considering the characteristics and purpose of ICG, we summarize the four most important uses of ICG in hepatobiliary surgery: (1) tumor visualization; (2) anatomical hepatectomy; (3) biliary tract imaging; and (4) liver transplantation evaluation (Fig. 1).

### Application of ICG fluorescence imaging technology in liver tumor visualization

ICG fluorescence imaging can be used for intraoperative visualization of most liver tumors, including hepatocellular carcinoma (HCC) and its metastases, HCC portal vein tumor thrombus, bile duct tumor thrombus, liver metastases from colon carcinoma, and other liver malignant and benign tumors^10,26,30–34^. ICG fluorescence imaging can detect tiny occult metastatic lesions, the smallest of which is about 1.5 mm.

HCC and liver metastases from colorectal carcinoma are the most common primary and secondary malignant tumors of the liver^35,36^. Intraoperative ICG fluorescence imaging could clearly show the liver lesions and achieve radical resection. Ishizawa *et al*.^10^ reported that ICG fluorescence imaging could identify HCC lesions with a sensitivity of 99%. In addition, 16 false-positive lesions were found, including regenerative nodules of liver cirrhosis, dysplastic nodules, bile duct hyperplasia, necrotic nodules, etc. The positive predictive value was 94%^10^. Abo *et al*.^37^ demonstrated that the ICG fluorescence imaging technique can be used to detect tumor thrombosis in the main portal vein of HCC by injection of ICG into the portal vein. With the ability to identify small and unrecognized HCC with high sensitivity, ICG fluorescence imaging can improve the accuracy of hepatectomy and surgical staging^10,26^. Boogerd *et al*.^38^ found that ICG fluorescence imaging had a sensitivity of 92%, which was much higher than other modalities, such as CT and MRI, in 26 patients with suspected HCC who were examined by different modalities. Interestingly, three of these metastases (12%) were identified only by ICG fluorescence imaging. The type of ICG fluorescent staining in HCC correlates with the degree of pathological differentiation^26^. Fluorescent staining of HCC can show three types of tumor staining such as complete, partial, and marginal ring staining. Highly differentiated and moderately differentiated HCC show both fully fluorescent and partially fluorescent phenotypes, whereas borderline fluorescent phenotypes are predominantly seen in poorly differentiated HCC with microvascular invasion and liver metastases^10,30^.

Colorectal cancer liver metastases (CRLM) do not take up ICG but are visualized due to ICG retention caused by compression of the peripheral hepatic bile ducts, which is manifested as marginal ring staining^26^. van der Vorst *et al*.^39^ revealed that ICG fluorescence imaging has good sensitivity for the identification of superficial CRLM lesions. In contrast, another multicenter retrospective study showed that the overall sensitivity of ICG fluorescence imaging in identifying CRLM lesions was 83%, with 100% sensitivity for CRLM lesions less than 8 mm from the liver surface^31^. Uchiyama *et al*.^40^ reported that ICG fluorescence imaging identified colorectal metastatic lesions with a sensitivity of 98.1%, compared with 88.5% for conventional imaging methods. Ishizawa *et al*.^26^ reported that the sensitivity and positive predictive value of ICG fluorescence imaging of liver metastases from colon cancer at the surgical specimen section was 100%. Peloso *et al*.^41^ reported that ICG fluorescence imaging combined with intraoperative ultrasound was superior for identifying CRLM lesions 3 mm or less in diameter, with a detection rate superior to that of preoperative CT or intraoperative ultrasound alone. While for lesions greater than 3 mm, the difference in the detection rate was not statistically significant. ICG fluorescence imaging could identify small liver metastases from colon cancer after neoadjuvant chemotherapy, which helps prevent local recurrence in superficial liver tissues^31,42^. Intraoperative ultrasound (IOUS) is currently regarded as the reference-standard method of guiding liver resections due to colon cancer metastasis^43^. Despite its indispensable advantage of real-time visualization, it also has several shortcomings, including the limited detected size (more than 3 mm), dependence on the skillful operator, and the presence of a superficial blind area about 1 cm under the liver surface. Previous studies have shown that the combined use of ICG and IOUS can overcome the limitations of each and identify a number of lesions that are not revealed by IOUS alone^40^. Thus, ICG fluorescence imaging helps detect small CRLM lesions on the liver surface or at the cutting edge, and the combined intraoperative ultrasound detects lesions 3 mm or less in diameter better than preoperative CT or IOUS alone. In conclusion, in terms of the intraoperative detection of liver metastases, the combination of ICG with IOUS may be an effective detection, especially in the presence of small metastases.

ICG fluorescence imaging can also be applied to detect other types of liver tumors such as intrahepatic cholangiocarcinoma^37^, hepatoblastoma^32^, neuroendocrine tumor liver metastases^44^, gastric cancer liver metastases^45^, pancreatic cancer liver metastases^46^, and melanoma liver metastases^47^. In addition, several benign liver lesions can be localized by ICG fluorescence imaging, including hepatic angiomyolipomas, cholangioadenomas, focal nodular hyperplasia (FNH), and cavernous hemangioma^38^. Harada *et al*.^47^ detected the role of ICG fluorescence in imaging cholangiocarcinoma (CCA) invasion. Hepatoblastoma, adenoma, FNH, epithelioid angiomyolipoma, etc., can uptake ICG to show tumor staining^32–34,49^. Most metastatic liver tumors show marginal ring staining, such as melanoma liver metastases, gastrointestinal mesenchymal tumor liver metastases, and pancreatic cancer liver metastases^47,50^. It has also been reported in the literature that liver metastases of gastric cancer and liver metastatic lesions of neuroendocrine tumors can take up ICG^44,45^. Fluorescence from the tumor or surrounding cholestasis can guide liver resection, and the presence of fluorescence in the section of the liver may signal tumor exposure^51^. To reduce the risk of positive resection margins, a suitable section should be determined to remove all tumor tissue with fluorescence^41^. ICG fluorescence imaging also has some limitations in identifying tumors, including limited fluorescence penetration, lack of specific targeting, boundary dispersion with inaccurate exhibition, and the presence of a certain false-positive rate^52^. Kudo *et al*.^26^ showed that ICG fluorescence imaging could only observe HCC and metastatic lesions within 8 mm of the liver surface or resection margin due to the limitation of near-infrared fluorescence penetration. Most liver tumors, including malignant liver tumors, secondary malignant liver tumors, and benign tumors, could be detected by ICG fluorescence imaging. Therefore, ICG fluorescence imaging lacks specific targeting^26,30,33^. A fluorescent contrast agent was diffusely distributed at the boundary between the liver tumor and normal liver parenchyma, which could not show the boundary accurately. This will lead to excessive resection of liver tissue during liver tumor resection and liver function damage^1,10^. Kose *et al*.^53^ reported that the recognition rate of ICG fluorescence imaging for superficial lesions was 95%, while the recognition rate of IOUS was 89%. For deep lesions, the recognition rate of ICG fluorescence imaging was only 4%, compared with 94% for IOUS. Therefore, intraoperative ultrasound is still needed for the localization of deep lesions. Although ICG fluorescence imaging has a high sensitivity and positive predictive value for tumor detection, with a median tumor identification rate of 87.4%, the median false-positive rate was 10.5% (0–31.3%)^23^. In particular, the false-positive rate of ICG fluorescence imaging in patients with cirrhosis is as high as 40%^10,26^. Due to the high false positive rate, the application of ICG in liver tumors will be limited to a certain extent. Despite these drawbacks, fluorescence molecular imaging has shown its potential to support surgical procedures. Improved image processing and enhancement would be desirable for the determination of cut-off values for resection margins and exclusion of false-positive signals. Among the factors leading to false-positives were cirrhosis, dysplastic nodules, short interval between ICG injection and surgery (i.e. <24 h), bile duct hyperplasia, necrosis, cysts, hemangiomas, and atypical non-malignant lesions^42,54^. Therefore, when new lesions are found by intraoperative ICG fluorescence imaging, careful judgment should be made. Combined with intraoperative ultrasound and contrast-enhanced imaging, as well as preoperative CT or MRI examination results, the nature of the lesion should be further identified. Needle biopsy or excisional biopsy should be performed if necessary. If a malignant tumor cannot be excluded, resection is recommended.

Nevertheless, clinical fluorescence imaging in human patients has been hindered by limited penetration depth in liver tissues. The detectable wavelength of ICG typically ranges between 750 and 810 nm, hampering deeper tissue imaging during hepatobiliary surgery^10^. To overcome the major limitation of near-infrared fluorescence image guidance for sensitive detection of deep lesions, the use of modified ICG contrast agents and multimodality imaging has been advocated. Recent studies have shown that extending fluorescence imaging into 900–1700 nm wavelength range, also known as near-infrared window II (NIR-II), could eliminate the deficiencies of NIR-I imaging^55,56^. Hu *et al*.^56^ reported 23 patients who underwent NIR-I/II fluorescence-guided surgical resection of primary and metastatic liver tumors and found that intraoperative NIR-II imaging provided higher tumor detection sensitivity. Combining the NIR-I/II spectral windows and suitable fluorescence probes might improve image-guided liver tumor resection. At present, fluorescent probes with molecular targeting functions are gradually being developed^57,58^. Wang *et al*.^58^ reported that the rapidly metabolized small molecule NIR-II fluorescent probe targeting HDAC6 protein can specifically target liver cancer, which has significant clinical transformation value. Zhang *et al*.^57^ reported the application of a nano-probe in the precise resection of HCC. During the operation, the lesions of HCC in the right hepatic lobe were found specifically on the NIR-II fluorescence image, and the clear edge was revealed, which improved the surgical accuracy^57^. In the future, it is a promising direction to study and design ICG fluorescence contrast agents with specific targeting to display the detailed edge of liver masses in real-time more accurately and make efforts to achieve accurate resection of liver tumors.

Recently, nuclear techniques have been particularly complementary to fluorescence navigation for tumor detection^59^. Contrast agents with radiolabeled and fluorescent dyes can inform surgery by targeting tumors. The design and preclinical validation of hybrid tracers of radionuclide and fluorescent ICG for surgical navigation tools for liver tumors are also promising directions in the future.

### Anatomical hepatectomy

**Figure 2:**
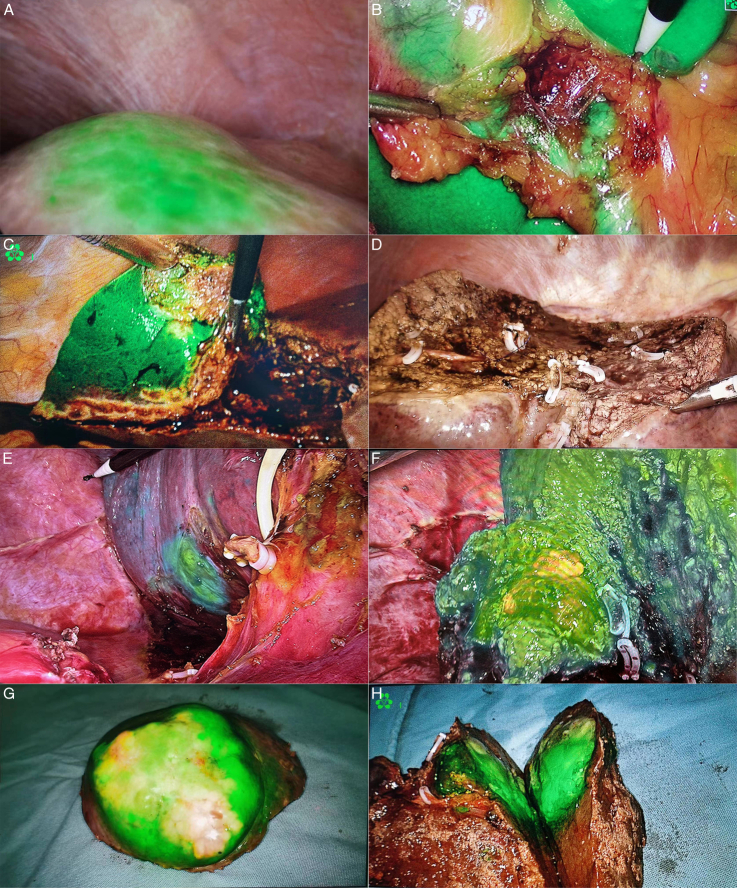
Clinical applications of indocyanine green (ICG) fluorescence imaging during liver resection. (A) visualization of hepatic segment VIII; (B) visualization of hepatic segment V; (C) ICG visualization during anatomical resection of hepatic segment Ⅵ; (D) liver section after hepatic segmentectomy; (E) visualization of the lesion between hepatic segments VI and VII; (F) hepatic segment VI hepatectomy with ICG; (G, H) the resected liver tumors.

**Figure 3:**
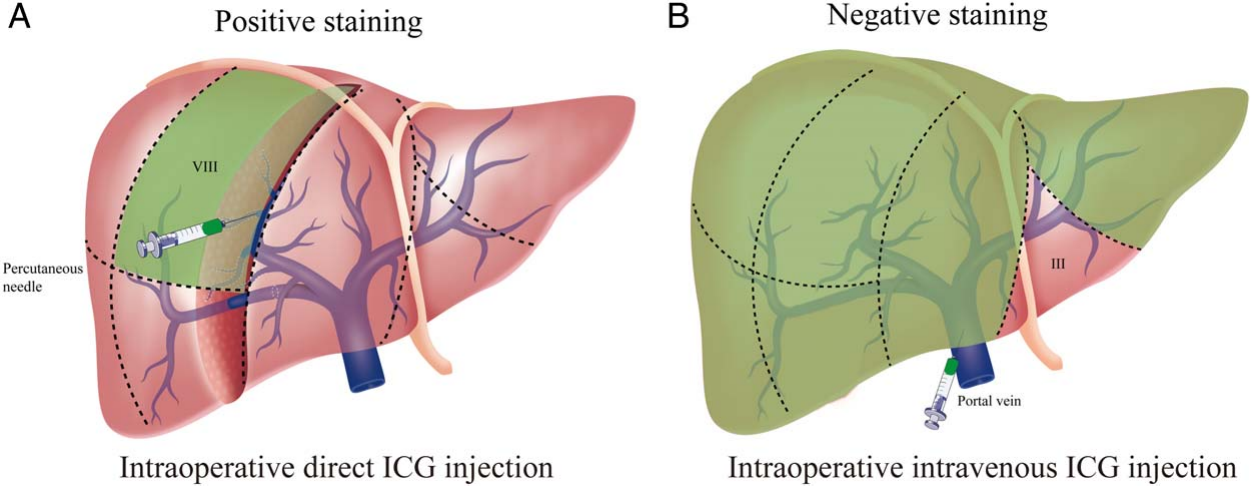
A schematic representation of indocyanine green (ICG) positive staining and negative staining. (A) anatomical hepatectomy of segment VIII with ICG-positive staining; (B) anatomical hepatectomy of segment III with ICG negative staining.

**Figure 4:**
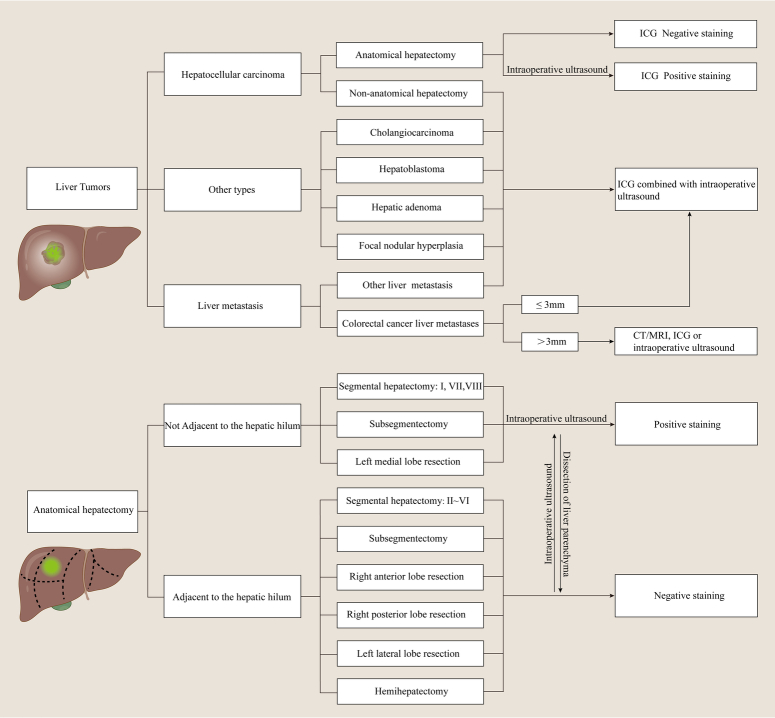
Practical scheme and implementation process for applications of indocyanine green (ICG) in liver resection. This practical scheme includes the application and clinical value of ICG fluorescence imaging technology in liver tumor staining and anatomical hepatectomy.

Anatomical hepatectomy refers to the complete resection of the hepatic parenchyma in the corresponding portal vein inflow and may have more oncological benefits than conventional hepatectomy^60^. The anatomical structure of the liver is complex, the vascular structure is variable, and the actual boundary between segments of the liver is staggered by a three-dimensional map (Fig. 2). Previous anatomical hepatectomy based on hepatic vascular occlusion and intraoperative ultrasound makes it difficult to achieve ‘anatomical’ resection in the true sense. In 2008, Aoki *et al*.^12^ reported that ICG fluorescence imaging was first used in open anatomical segmentectomy or subsegmentectomy, and ultrasound-guided puncture was used to inject ICG into the target portal vein to stain the corresponding drainage area, with a success rate of 94.3%. In 2012, Ishizawa *et al*.^13^ reported laparoscopic ICG fluorescence-guided anatomical hepatectomy and proposed two strategies for ICG fluorescence staining: positive staining and negative staining^61^. Compared with traditional technology, ICG fluorescence visualization can provide real-time three-dimensional boundaries of the liver surface and parenchyma, and can also clearly identify and display the intersegmental plane of the liver, which is better to guide anatomical hepatectomy^62,63^. The positive staining method was to directly puncture the target portal vein, inject an appropriate amount of ICG, and show the corresponding portal vein drainage area. With the help of the positive staining method, the boundary of the outflow vein watershed could be marked before the liver parenchyma was separated, and the staining boundary was stable. Compared with the methylene blue technique, positive staining is a major advance in identifying liver segments before parenchymal dissection. The positive staining method was suitable for the staining of the S7 and S8 segments of the liver, where part of the liver parenchyma needs to be cut to expose the hepatic pedicle^25^. For negative staining, ICG was injected into the peripheral vein after blocking the hepatic pedicle of the target liver segment to prevent blood from entering the tumor-related segment^24^. The negative staining method is suitable for the resection of liver segments adjacent to the hepatic hilum and easy to dissect, such as hemihepatectomy, right anterior liver resection, right posterior liver resection, and partial liver resection (S2–S6)^64^. Positive staining is recommended for open surgery and resection of individual segments, which allows adjustment of staining intensity and area to better identify the intersegmental plane (Fig. 3). In clinical practice, for hemi-liver or right anterior or right posterior combined liver segments, both positive staining and negative staining can guide liver resection. Therefore, the ICG fluorescence staining strategy should be selected according to the location of the tumor and the anatomy of the corresponding portal vein or Glissonean pedicle in anatomical hepatectomy (Fig. 4).

As for hemihepatectomy, generally, the Glissonean pedicle of the left and right halves of the liver is easily exposed at the first hepatic hilum, so the negative staining method is relatively convenient and easy to operate. However, during ICG fluorescence staining, attention should be paid to vascular variations, including extrahepatic artery blood supply, small branches of Glissonean in the hepatic hilar region, gallbladder vein reflux, and the presence of arteries and portal vein communication between the left and right liver^65–67^. Takeuchi *et al*.^65^ performed CT angiography of extrahepatic arteries in 23 patients with liver tumors and found that when the proper hepatic artery was temporarily blocked, the right inferior phrenic artery could immediately be seen to supply blood to the liver in 85% of the cases. Sugita *et al*.^66^ reported that CT examination during angiography of the gallbladder in 27 patients revealed that the gallbladder vein converged into the intrahepatic portal vein or the hepatic sinusoids in all cases and that its main reflux area was S4 or S5 of the liver. Tohma *et al*.^67^ performed hepatic arteriography on 11 patients and found that there were communicating branches between the left and right hepatic arteries in all cases. Using the Laennec membrane approach and the Glissonean pedicle first approach, the right anterior area, the right posterior area and the left lateral area of the Glissonean pedicle can be exposed anatomically, and positive or negative staining can be carried out^68^.

Li *et al*.^69^ reported that 26 cases of laparoscopic right posterior hepatectomy were successfully performed under the guidance of ICG fluorescence imaging with the Glissonean pedicle first approach, and achieved good results. Zeng *et al*.^70^ performed a successful laparoscopic right posterior hepatectomy using the Laennec membrane approach. Deng *et al*.^71^ performed a successful laparoscopic right anterior lobectomy using ICG fluorescence imaging. In a word, the Glissonean pedicle first approach and the Laennec membrane approach have played an important role in ICG fluorescence imaging laparoscopic hepatectomy.

During hepatic segmentectomy, ICG fluorescence imaging plays an important guiding role. The Glissonean pedicle of hepatic segments such as S2–S6 is adjacent to the hepatic hilum, so the corresponding Glissonean pedicle can be easily dissected and exposed through the Laennec membrane. Lin *et al*.^72^ reported that 12 cases of laparoscopic anatomical S2/3 resection were successfully performed by ICG fluorescence image-guided TICGL technique (temporary inflow control of the Glissonean pedicle). Seven patients underwent S2 resection, and five patients underwent S3 resection. Wan *et al*.^73^ reported the effectiveness of the Glissonean pedicle first approach combined with negative ICG fluorescence staining in the resection of hepatic S3 and S4 segments. Since then, the application of ICG fluorescence imaging in hepatic S4 segment resection and hepatic S5 segment resection has also been verified and achieved good results^74,75^. In addition, Mizuno *et al*.^76^ reported the navigation role of ICG-negative staining combined with Laennec membrane and Glissonean pedicle approach in hepatic S6 segment resection, providing a new idea for hepatic segment resection alone. It is worth noting that the Glissonean pedicle of the S6 segment (G6) needs to be preserved when the S7 segment is resected because the S7 Glissonean pedicle (G7) emanates from the beginning of the Glissonean pedicle of the right posterior branch separately from G6. Minami *et al*.^77^ reported that in about 45% of cases, the right posterior Glissonean pedicle is a two-branch type, in which case G7 is easy to be separated and blocked. In this case, there may be multiple branches of the Glissonean pedicle in S6 or S7, and G7 is far from the beginning of the right posterior branch. When it is difficult to dissect G7 outside the liver, part of the liver tissue needs to be dissected. The S8 Glissonean pedicle (G8) has more variations in number and alignment and is deeply located. It is necessary to master the anatomical characteristics of the blood vessels in the right anterior area, pay attention to protect the Glissonean pedicle of S5 and cut part of the liver tissue in the hepatic hilum to expose the S8 Glissonean pedicle. The traditional method is to split the liver parenchyma through the median fissure and find the liver S8 Glissonean pedicle in the liver parenchyma through the middle hepatic vein. The latest research found that intraoperative ultrasound combined with ICG fluorescence imaging-guided puncture of portal vein branches can mark the limit of S8 without additional dissection of liver parenchyma, which has obvious advantages^25^. For the whole caudate lobe of S1, the positive staining method can be more convenient, but there are few literature reports.

Subsegmentectomy is mostly used for S1, S4, S8, etc., which can be performed by positive or negative staining^60,78^. For example, the Glissonean pedicle of left caudal lobe G1L, right caudate process G1c, and S4b are convenient to dissect, and negative staining can be used directly. The S4a Glissonean pedicle and the ventral and dorsal Glissonean pedicle of S8 can be found after splitting the liver parenchyma and can be negatively or positively stained^79,80^ (Fig. 3).

ICG fluorescence imaging also has application value in hepatic echinococcosis surgery. The biggest feature of hepatic echinococcosis is invasive growth, which can not only destroy the intrahepatic bile duct structure but also invade distant organs through metastasis. Because alveolar echinococcosis does not take up ICG, hydatid lesions show ‘negative staining’ after ICG injection in the peripheral vein during alveolar echinococcosis surgery^81^. When ICG was injected intravenously during the operation, there was a clear boundary between the fluorescence of the hepatic cystic echinococcosis lesion and the surrounding liver tissue without fluorescent staining in the hepatic cystic echinococcosis lesion. Surgeons can use this space to remove the external capsule, which can improve the complete removal rate of the external capsule^82^. Both hepatic alveolar echinococcosis and cystic echinococcosis grow along the biliary tract. ICG fluorescence imaging is helpful to detect intraoperative bile leakage in the liver section and to reduce postoperative complications such as bile leakage. Using ICG fluorescence visualization, the relationship between hydatid lesions and intrahepatic bile ducts can be displayed in real time to avoid damage to the main bile ducts.

In a word, for liver segments or subsegments, the negative staining method was used when the Glissonean pedicle was adjacent to the hepatic hilum, and the positive staining method was used when the Glissonean pedicle was deep (Fig. 4). ICG fluorescence imaging technology has important clinical value and application prospects in anatomical hepatectomy and hepatic echinococcosis surgery, which is helpful to improve the accuracy of liver surgery. In the future, more multicenter randomized controlled trials are needed to determine the optimal use time and dose of ICG in anatomical hepatectomy. Clinical hepatobiliary surgery requires a lower ICG dose, a longer duration of real-time display for liver segments, and a higher image brightness of fluorescence imaging. In addition, how to realize the quantification of the intensity of the color in terms of blood supply and show the hallmark of greenness grading for adequate supply will be a promising research direction for anatomic hepatectomy. Determining the ICG dose in the context of different degrees of cirrhosis is also a point to be explored in the future.

### Application of ICG fluorescence imaging technology in biliary tract imaging

#### Application of ICG fluorescence imaging technology in LC

**Figure 5:**
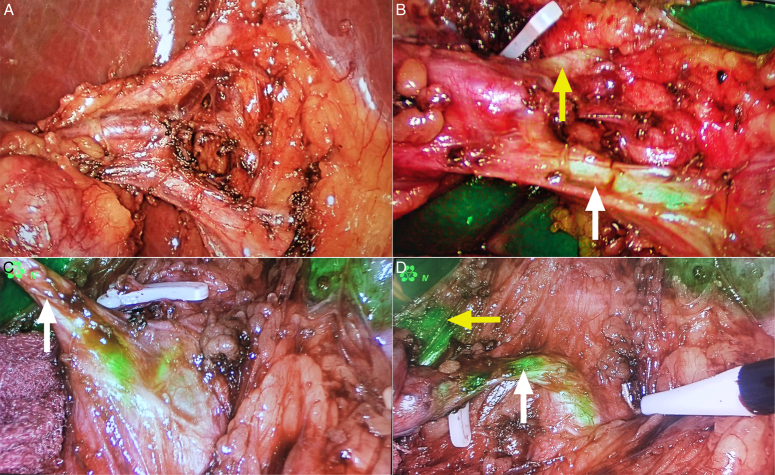
Clinical applications of indocyanine green (ICG) fluorescence imaging during laparoscopic cholecystectomy. (A) original view of cystic duct variation; (B–D) ICG fluorescence imaging of cystic duct variation.

Laparoscopic cholecystectomy (LC) is the standard surgical treatment for gallstones, and the incidence of bile duct injury during LC is 0.2% to 1.5%^83^. There are many reasons for intraoperative bile duct injury, the most common of which is insufficient recognition of the bile duct, especially when the area is combined with inflammation, edema, obesity, or bile duct variation^84,85^. Intraoperative cholangiography can help the operator to better identify the biliary anatomy and avoid biliary injuries (Fig. 5). In 2009, Ishizawa *et al.* ^19^ first reported the acquisition of biliary fluorescence imaging in LC by intravenous injection of ICG, which reduced the incidence of bile duct injury during LC. They also found that the concentration of ICG in the biliary tract reached the peak 2 h after intravenous injection, so intravenous injection of 2.5 mg ICG 1 h before LC was safer and more convenient than transcystic cannulation for biliary tract imaging^18,19^. Schols *et al*. reported that ICG fluorescence cholangiography(ICG-FC) showed a common bile duct in 83% of patients and a cystic duct in 97%. In contrast, conventional imaging revealed a common bile duct in only 73% of patients and a cystic duct in 97%^86^. Studies have shown that fluorescence imaging can improve the recognition rate of extrahepatic bile duct structures compared with white light mode imaging^87,88^. ICG-FC can also clearly show extrahepatic biliary structures^87^. During elective LC, the common bile duct was identified more quickly by using ICG-FC (22 min) than the conventional method (32 min)^89^. To reduce the risk of bile duct injury, techniques to enhance proper identification of the anatomy are required. Proposed by Strasberg in 1995^90^, the Critical View of Safety (CVS) technique is the gold standard to conduct a safe cholecystectomy with identification of the cystic duct^91^. An international multicenter randomized controlled trial conducted by Bos *et al.* ^92^ found that the use of NIR ICG assisted LC achieved statistically significant earlier recognition of critical view of safety and cystic duct visualization compared to the non-ICG use LC group (19 vs. 23 min for CVS). The time to identify cystic duct in the NIR ICG group was shorter than non-ICG use group (6 vs. 13 min). In addition, Broderick *et al.* ^93^ analyzed 1389 patients undergoing cholecystectomy and found that the operation time of the ICG group and the non-ICG group was 75.57 min and 104.9 min, respectively. Compared with traditional LC, ICG-FC assisted LC significantly reduced the operation time and average hospital stay^93,94^. Other series have shown that ICG-FC is feasible, safe, and shortens procedure time in laparoscopic and robotic cholecystectomy^95–97^. In contrast, ICG-FC can identify the cystic duct in all examined patients without dissecting the Calot’s triangle^18^. A recent study has shown that the ability of ICG fluorescence imaging to identify aberrant bile ducts is significantly better than that of white light mode^87^. ICG can identify cystic duct variation and avoid iatrogenic bile duct injury (Fig. 5).

Due to the limited penetration depth of near-infrared imaging, the recognition rate of bile ducts in patients with obesity, inflammation, and biliary re-operation is decreased. Some studies have confirmed that ICG-FC has a positive effect on obesity (BMI>26.35 kg/m2) or the extrahepatic bile duct of the Calot’s triangle cannot be visualized in patients with chronic cholecystitis, probably because ICG-FC can only penetrate human tissue to a depth of about 10 mm^98^.

In conclusion, ICG fluorescence imaging can better visualize the biliary tract in LC, thereby reducing the potential risk of bile duct injury, improving the safety of LC, and reducing intraoperative radiation cholangiography. ICG is of great significance for young surgeons who are initially performing LC and for patients with extrahepatic biliary tract variants.

#### Application of ICG fluorescence imaging technology in hepatolithiasis surgery

Hepatolithiasis is a complex condition that poses challenges and difficulties in surgical treatment. ICG fluorescence imaging in complex hepatolithiasis surgery can obtain real-time biliary imaging, reduce the operation time, and avoid the risk of bile duct injury. In the surgical treatment of hepatolithiasis, ICG fluorescence technology plays a real-time navigation role for precise hepatectomy. Ishizawa *et al.* ^18^ reported that ICG-FC can identify all stones in the cystic duct, which helps determine the cut-off point of the cystic duct and avoid missing stones in the cystic duct. However, small stones in the common bile duct may be obscured by the strong ICG fluorescence signal that surrounds them. In addition, due to the limited penetration of near-infrared fluorescence imaging, the bile duct behind the pancreas cannot be developed, so ICG-FC cannot completely exclude common bile duct stones^53^. When hepatectomy is needed for the treatment of hepatolithiasis, the demarcation line of the target liver segment is often determined according to the inferior vena cava, gallbladder fossa, liver fissure, and other landmark structures, which cannot truly achieve navigation hepatectomy. On this basis, ICG fluorescence imaging technology could be used to adjust the surgical plane in real-time and achieve accurate hepatectomy of the hepatolithiasis segment^99^. Huang *et al.* ^100^ reported three-dimensional visualization technology combined with fluorescence imaging may have significant benefits in surgical safety, reduced intraoperative bleeding, and decreased stone residue during hepatectomy for hepatolithiasis. Recent study has shown that ICG peripheral venography-assisted hepatectomy is superior to conventional hepatectomy for the treatment of hepatolithiasis in terms of blood loss, hospital stay, and postoperative inflammatory markers^99^.

In clinical application, patients with complex hepatolithiasis surgery, previous hepatolithiasis surgery history, or other upper abdominal surgery history need to undergo second hepatobiliary surgery, and it is difficult to identify the bile duct during operation due to upper abdominal adhesion or scar. The application of ICG fluorescence bile duct imaging helps to identify bile duct structures in real-time, detect bile duct stones, avoid damage to blood vessels or surrounding tissues such as the gastrointestinal tract, and shorten the time to find and isolate the bile ducts^99^. Therefore, only using ICG-FC may miss small bile duct stones or pancreatic segment stones. It is still necessary to combine with traditional methods to better apply ICG imaging technology in hepatolithiasis surgery.

#### Application of ICG fluorescence imaging technology in bile duct injury and bile leakage

Bile duct injury(BDI) and bile leakage are common complications of biliary tract surgery, which seriously threaten the prognosis of patients undergoing biliary tract surgery. Image-guided surgery can potentially reduce the risk of BDI. The characteristics of ICG excretion through the biliary tract in vivo can be used for intraoperative visualization of the extrahepatic bile duct to reduce iatrogenic BDI and to identify intraoperative bile leakage. Currently, X-ray intraoperative cholangiography (IOC) is the gold standard method for intraoperative identification of biliary anatomy. However, IOC is underutilized owing to significantly increased operating time, added cost, potential risk of BDI associated with IOC, and radiation exposure^101,102^. In difficult biliary anatomy surgery, IOC is extremely difficult to perform in patients with unascertainable extrahepatic bile duct due to the extensive adhesion of the surgical field and the unclear identification of the biliary tract. Lehrskov *et al*.^103^ demonstrated the non-inferiority of ICG-FC as a non-invasive alternative of IOC for identifying the critical junction between common hepatic, cystic, and common bile duct. Besides, the visualization rates of the cystic–common bile duct junction in ICG-FC were higher than that in IOC (98.15% vs. 96.15%), and ICG-FC was faster than IOC (107.8 s vs. 1355.77 s)^104^.

For patients with complex biliary tract variations, ICG-FC can replace conventional imaging cholangiography to reduce the incidence of biliary tract injury. Maker *et al*. reported that ICG-FC is more effective in the identification and visualization of extrahepatic biliary than IOC in difficult biliary tract cases^105–107^. This simple technique may become standard practice for avoiding bile duct injury during LC, replacing radiographic cholangiography^18,108^. In our center, we found the complex extrahepatic biliary variation in the right posterior segment with the help of ICG Fluorescence Imaging (Fig. 5). It revealed that the bile duct in the right posterior segment opened in the cystic duct and shared a common trunk with the cystic duct before entering the liver.

In recent years, more and more studies have reported cases of bile leakage identified by ICG fluorescence imaging during cholecystectomy^109,110^. Due to the limited penetration depth of near-infrared fluorescence, the intrahepatic bile duct cannot be seen on the surface of the liver after the injection of ICG into the vein or bile duct. However, with the liver parenchyma being separated, the intrahepatic bile duct located on the surface of the Glissonean pedicle can be seen, which is helpful to properly handle the intrahepatic bile duct and avoid bile leakage^111^. Therefore, the application of ICG imaging can avoid biliary tract injury and bile leakage. Daskalaki *et al*.^112^ reported that 184 cases underwent ICG-FC, and the successful visualization rates of the cystic duct, common bile duct, and common hepatic duct were 97.8%, 96.1%, and 94%, respectively, without BDI. Pesce *et al*.^113^ suggested that ICG fluorescence imaging could significantly improve the detection sensitivity of important anatomical structures of the biliary tract and help prevent BDI in operations in which the biliary structures in the hepatic hilar area were not displayed, such as atrophic gallbladder, gallbladder re-operation or bile duct surgery. Sakaguchi *et al*.^114^ reported earlier cases of ICG fluorescence imaging in 27 patients with bile leakage after hepatectomy. Kaibori *et al*.^115^ used the same technique to treat 52 patients who underwent hepatic resection, and no bile leakage occurred after the operation, while the incidence of bile leakage in the control group was 10%. Recently, a randomized controlled trial of emergency LC found that routine use of ICG improved surgical safety in emergency LC and reduced the incidence of BDI and bile leakage^116^. ICG fluorescence imaging can provide adequate biliary structure under near-infrared light windows, increase resolution and penetration depth, and may be an effective option to improve the safety of biliary surgery in difficult cases^117^.

In conclusion, ICG fluorescence imaging technology can help avoid intraoperative injury and reduce the risk of bile leakage during biliary surgery. However, it is not clear whether BDI or cholangitis affect intraoperative ICG-FC. Prospective multicenter randomized clinical trials are needed to determine whether ICG fluorescence imaging can reduce the incidence of BDI and to determine the optimal timing and dose of ICG.

### Application of ICG fluorescence imaging technology in liver transplantation

There are few reports on the application of ICG fluorescence imaging in living donor liver transplantation (LDLT), mainly focusing on donor-recipient biliary anastomosis imaging and the evaluation of donor liver quality in liver transplantation^118^. In 2010, Mizuno and Isaji^119^ reported for the first time that ICG was injected through the cystic duct during open left hemihepatectomy, and the left hepatic duct and right posterior bile duct were visible under fluorescence, which could be guided to determine the bile duct line in real-time. In 2017, Hong *et al*.^120^ reported that 0.05 mg/kg ICG was injected intravenously 30–60 min before exposure to the hepatic hilar plate during laparoscopic living donor liver resection. The optimal bile duct incision line was determined under direct visualization when the right and left hepatic ducts, caudate lobe, and variant bile ducts were distinguished. Therefore, compared with traditional intraoperative angiography, ICG fluorescence imaging technology can observe the bile duct from different angles, which is helpful in understanding the three-dimensional spatial relationship between the bile duct and the surrounding structures of the hepatic hilar plate and determining the optimal bile duct tangent line. ICG fluorescence imaging technology is also helpful in determining the plane of donor liver disconnection during liver transplantation. In 2015, Kim *et al*.^121^ reported that ICG fluorescence imaging could accurately display the left and right hemi-liver planes during living donor hepatectomy, shorten the operation time, and reduce liver function damage. Li *et al*.^122^ reported the application of ICG-negative staining in laparoscopic S2 single-segment living donor liver resection, which was helpful in determining the S2 and S3 sections of the liver (Fig. 1). Therefore, ICG fluorescence imaging can show the boundaries between hemi-liver and individual liver segments and facilitate donor liver separation. Assessment of bile duct blood supply during liver transplantation is usually performed by assessing the performance of the recipient and donor bile duct stump. There are few studies on the application of ICG fluorescence imaging in the evaluation of bile duct blood supply in liver transplantation. Coubeau *et al*.^123^ used ICG fluorescence angiography to evaluate bile duct vascularization, and intraoperative fluoroscopy was performed after the injection of ICG (0.25 mg/kg) through a central catheter. By ICG evaluation, the ischemic area was identified and resected. Conventional percutaneous cholangiography at 6 months after operation showed no stenosis or leakage. Primary graft dysfunction (PGD) is the main cause of death after liver transplantation. Figueroa*et al*.^124^ first reported the use of ICG as a predictor of graft function in LDLT.

In conclusion, ICG fluorescence imaging is a multifunctional tool with many useful applications during LDLT, including liver function assessment, determination of bile duct resection line, determination of donor liver transection plane, assessment of bile duct blood supply, and prediction of graft function. However, further studies and clinical trials are needed to verify and standardize its routine use in liver transplantation.

## Summary and prospect

In summary, ICG fluorescence imaging technology has important clinical value and application prospects in liver tumor staining, anatomical hepatectomy, visualization of intrahepatic and extrahepatic bile ducts, and living donor liver transplantation, which is helpful in improving the accuracy of liver surgery and oncological efficacy (Fig. 4). However, current ICG fluorescence imaging technology also has some limitations, including limited tissue penetration, insufficient sensitivity, poor specificity, lack of specific targeting, boundary dispersion with inaccurate exhibition, and the presence of a certain false-positive rate. Further clinical studies and techniques are needed to improve the ICG fluorescence imaging technology. In the future, it is necessary to design a specific targeted ICG fluorescence contrast agent or fluorescent molecular probe to more accurately display the detailed edge of liver tumors in real-time. The use of ICG in hepatobiliary surgery needs to have the advantages of lower doses, longer duration of real-time tumor display, higher brightness of fluorescence imaging, and more accurate tumor edge. The application of ICG fluorescence imaging technology in hepatobiliary surgery is still in the exploratory stage, and this review summarizes the clinical experience of a small number of centers. In addition, the sensitivity of different fluorescence imaging devices is different, and the dose of ICG is also different. Therefore, the wide application of ICG fluorescence imaging in the field of hepatobiliary surgery needs prospective clinical studies to provide a higher level of evidence.

## Ethical approval

Not applicable.

## Consent

This article is a review, and no informed consent was obtained from the patient.

## Source of funding

This work was supported by the Natural Science Fund for Outstanding Young Scholars of Hunan Province (No. 2024JJ2037), Natural Science Foundation of Hunan Province (No. 2023JJ40370), National Natural Science Foundation of China (No. 22374045 and No. 82303186), and the Project of Hunan Provincial Health Commission (Z2023031).

## Author contribution

J.Z., Y.L., and S.L.: study concept and design; J.Z. and Z.T.: drafting of the manuscript; S.L. and Y.L.: critical revision of the manuscript for important intellectual content; S.L.: administrative, technical, or material support; J.Z., Z.T., and B.S.: preparing figures; Y.L. and S.L.: study supervision. All authors have made a significant contribution to this study and have approved the final manuscript.

## Conflicts of interest disclosure

All authors have no conflicts of interest related to this publication. No benefits in any form have been received or will be received from a commercial party related directly or indirectly to the subject of this article.

## Research registration unique identifying number (UIN)

https://classic.clinicaltrials.gov/ct2/show/NCT05546619. ClinicalTrials.gov Identifier: NCT05546619.

## Guarantor

Sulai Liu, deputy chief surgeon of the Department of Hepatobiliary Surgery of Hunan Provincial People’s Hospital, Director of Central Laboratory of Hunan Provincial People’s Hospital.

## Data availability statement

Data during the current study will be made available from the corresponding author on reasonable request. All data generated or analyzed during this study are included in this manuscript.

## Provenance and peer review

Not commissioned, externally peer-reviewed.

## Assistance with the study

Not applicable.

## Presentation

Not applicable.
